# A Rare Case of a Retroperitoneal Abscess Due to Trichosporon spp. in an Immunocompetent Patient

**DOI:** 10.7759/cureus.55656

**Published:** 2024-03-06

**Authors:** Tamer Boudriiya, Imane Zouaoui, Salim Lachkar, Mostaine El Mamoune, Sarra Aoufi

**Affiliations:** 1 Central Laboratory of Parasitology and Mycology, Ibn Sina University Hospital, Faculty of Medicine and Pharmacy, Mohammed V University, Rabat, MAR; 2 Urology A, Ibn Sina University Hospital, Faculty of Medicine and Pharmacy, Mohammed V University, Rabat, MAR

**Keywords:** voriconazole, immunocompetent, invasive trichosporonosis, abscess, trichosporon

## Abstract

This report discusses a rare case of retroperitoneal infection caused by *Trichosporon spp.* in a 68-year-old immunocompetent woman following a nephrectomy. *Trichosporon spp.* was identified through meticulous mycological examination. This case challenges the typical association of *Trichosporon* infections with immunocompromised patients, emphasizing its potential pathogenicity in immunocompetent individuals. The importance of accurate identification, especially in postoperative infections and broad-spectrum antibiotic contexts, is highlighted, emphasizing the need for a thorough diagnostic approach in such cases.

## Introduction

Invasive fungal infections (IFI) represent a significant cause of mortality in vulnerable patient populations, with *Trichosporon spp*. ranking as the third most common causative agent of IFI, following *Candida spp*. and *Aspergillus spp*. [1].

Retroperitoneal infections, albeit rare, present a diagnostic challenge due to their insidious nature and the frequent absence of characteristic clinical signs. These infections are primarily secondary to renal conditions. While Gram-negative bacilli are currently the most frequently implicated pathogens, sporadic cases of fungal origin are reported, particularly in neutropenic patients.

We present a case involving an immunocompetent patient who exhibited pyonephrosis due to a kidney stone, necessitating a nephrectomy. Subsequently, she developed a retroperitoneal abscess caused by *Trichosporon spp*..

## Case presentation

A 68-year-old patient with a history of arterial hypertension and left nephrolithiasis, who had experienced multiple episodes of pyelonephritis treated with antibiotics, presented to the emergency department with left pyonephrosis. Further characterization of the nephrolithiasis revealed a pelvic kidney stone measuring 39 mm, along with three additional stones located in the upper calyx, measuring 14 mm, 15 mm, and 9 mm, respectively. She underwent drainage through percutaneous nephrostomy and received antibiotic treatment, leading to substantial clinical improvement. The cytobacteriological examination of pelvic urine returned negative results. Subsequent renal scintigraphy indicated that renal function was adequately sustained, with 95% attributed to the right kidney and 5% to the left kidney. Consequently, the patient underwent a left total nephrectomy via transverse lumbotomy.

**Figure 1:**
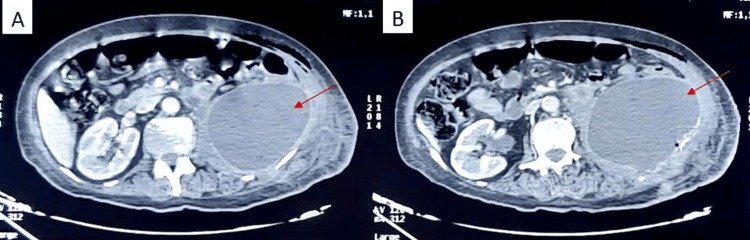
Axial CT images of the abdomen (A,B) showing a left retroperitoneal collection.

**Figure 2:**
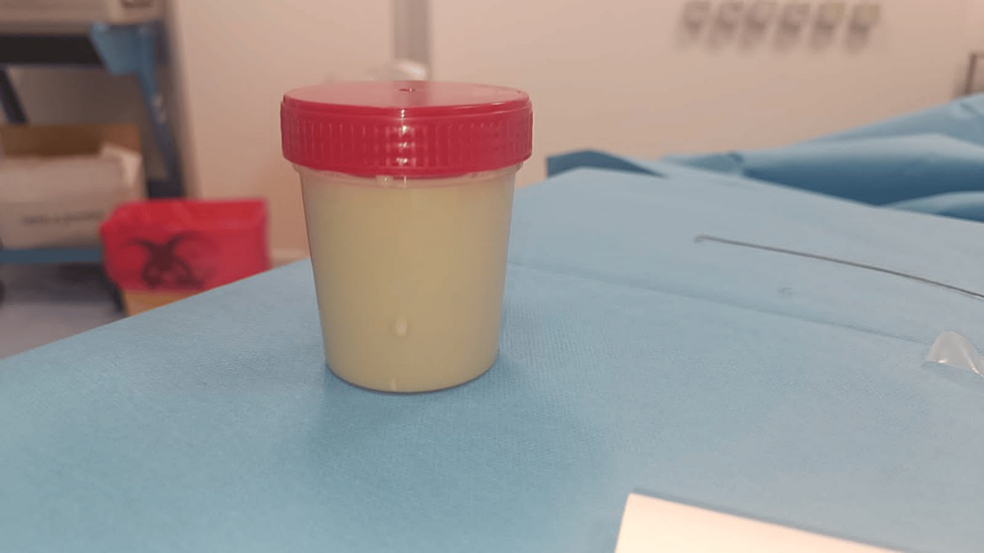
Vial containing pus from surgical drainage.

Two weeks after the operation, the patient presented to the emergency department with complaints of pain in the left lumbar fossa, fever, and a general decline. Laboratory tests showed leukocytosis at 11,000 cells/mm^3^ and a C-reactive protein concentration of 102 mg/l. Given the postoperative context, a CT scan was performed, revealing a large fluid collection in the left renal bed, indicative of a nosocomial postoperative abscess (Figure 1). The patient underwent immediate surgical drainage, yielding approximately 300 cc of pus (Figure 2). The pus was sent for microbiological analysis, and the patient received antibiotic therapy covering nosocomial pathogens.

Despite antibiotic treatment, clinical and biological parameters did not improve, suggesting the ineffectiveness of the antibiotic therapy. The pus sent to the parasitology and mycology laboratory was cultured on Sabouraud media and incubated at 37 °C. Direct examination revealed mycelial filaments with arthroconidia.

**Figure 3:**
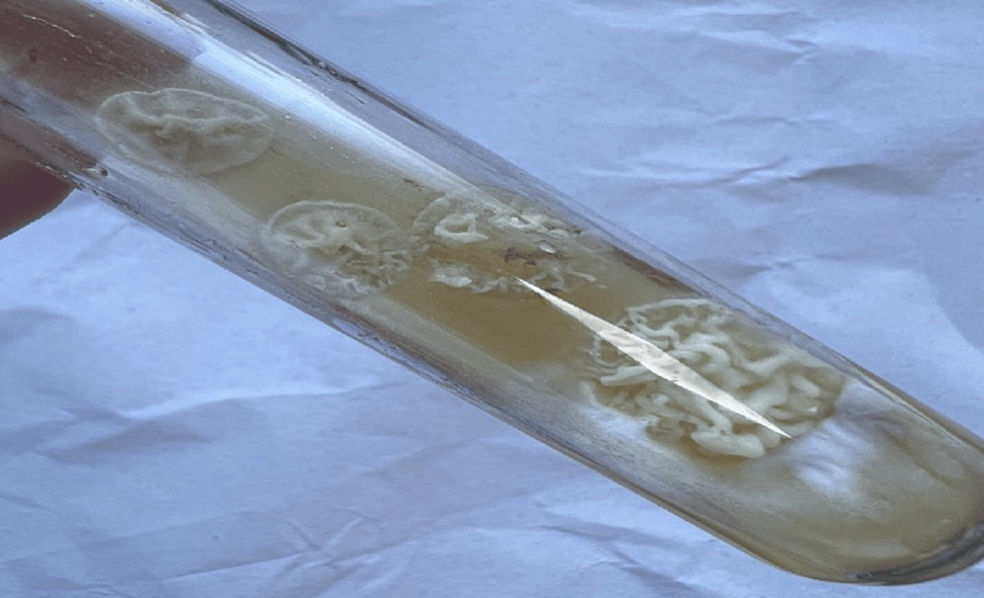
Cerebriform colonies on Sabouraud medium.

**Figure 4:**
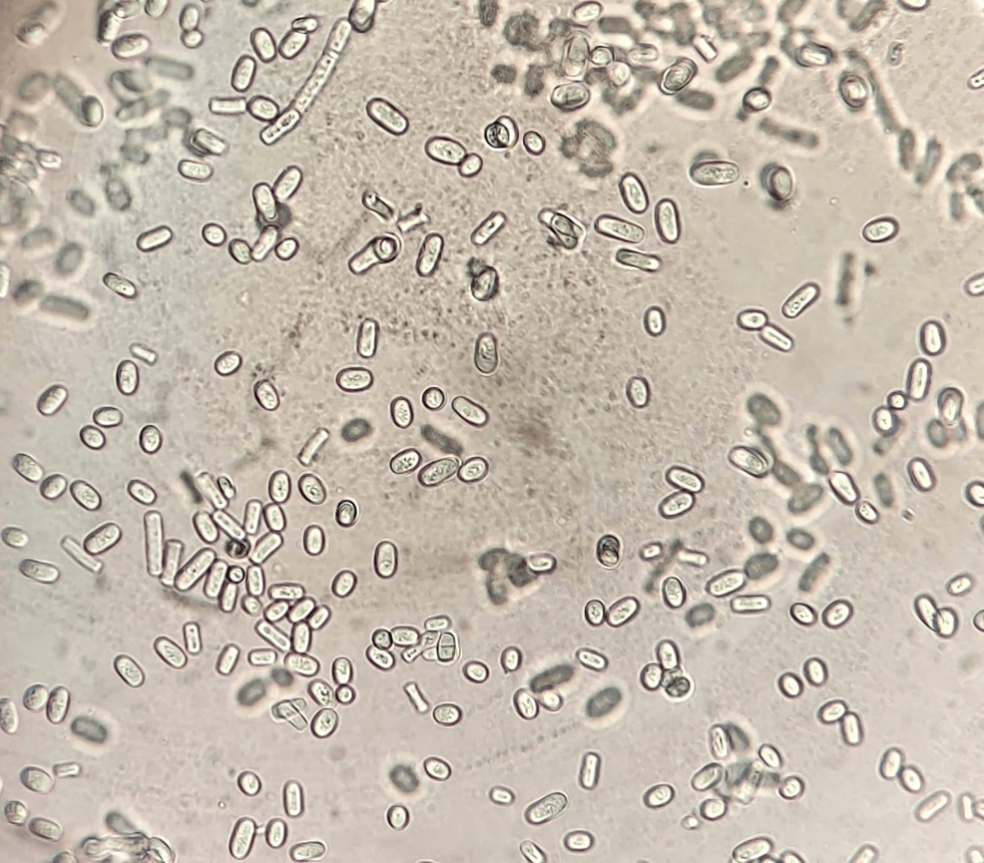
Microscopic direct examination of the culture revealing arthroconidia and blastoconidia (X400).

After 48 hours, beige and cerebriform colonies were observed in culture (Figure 3). Microscopic examination of these colonies showed hyaline and septate mycelial filaments with numerous blastoconidia and arthroconidia (Figure 4). The urea test was positive, and the biochemical identification gallery (AuxaColor BioRad®) favored *Trichosporon spp..*

**Figure 5:**
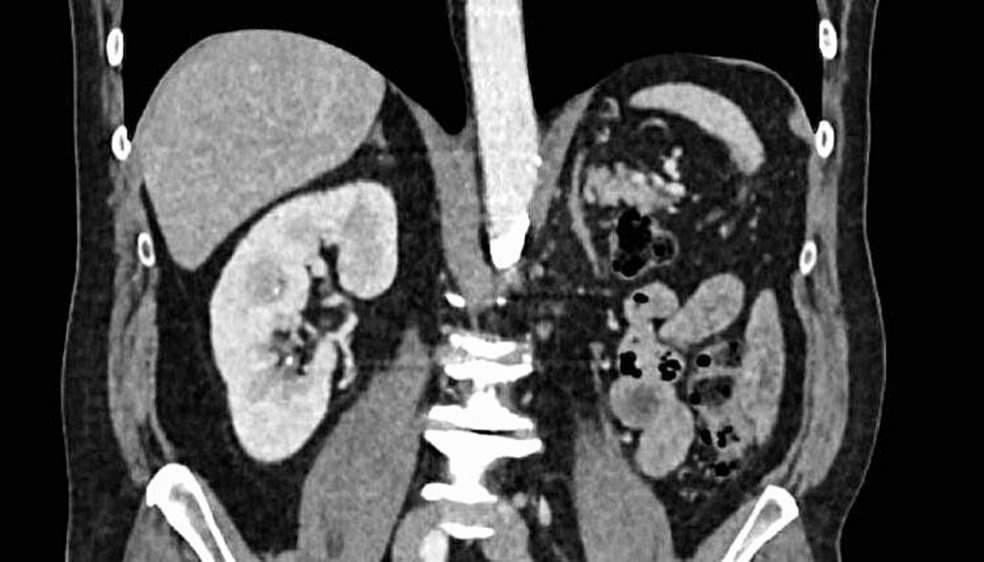
Coronal CT scan showing a well-healed left nephrectomy bed with no evidence of recurrent abscess.

The patient was prescribed voriconazole at 800 mg/day on the first day, followed by 400 mg/day for three months, resulting in significant improvement in both clinical and biological parameters. Follow-up imaging showed a complete regression of the retroperitoneal collection (Figure 5).

## Discussion

Over the past 30 years, the incidence of IFI has significantly increased in patients with cancer and severe burns, organ transplant recipients, and those treated with immunosuppressive agents, thereby becoming one of the leading causes of death. These infections are primarily dominated by candidiasis and aspergillosis, but cases of trichosporonosis have also seen a rise and are increasingly reported worldwide, causing severe and sometimes fatal infections in immunocompromised individuals, as well as occasionally in immunocompetent patients [2-4].

Approximately 50 species of the *Trichosporon* genus have been characterized, among which 16 are associated with diseases in humans. Guého et al. identified species that show significant adaptation to the human body, namely, *Trichosporon* *asahii*, *Trichosporon asteroides*, *Trichosporon* *cutaneum*, *Trichosporon inkin*, *Trichosporon mucoides*, and *Trichosporon ovoides*. These six species remain the most clinically relevant to date [4].

The pathogenicity of *Trichosporon spp.* is attributed to the presence of certain virulence factors that promote its growth and dissemination and enable it to evade the immune system. Among these factors are (a) the ability to adhere to implanted devices and form biofilms. This biofilm facilitates surface invasion by evading the host's immune response and the effects of antifungal medications (b) the secretion of enzymes (proteases and phospholipases) that disrupt the host's cell membrane proteins, thereby promoting fungal invasion, and (c) the expression of glucuronoxylomannan on the cell membrane, which inhibits the phagocytosis of this yeast by monocytes and neutrophils [5].

Yeasts of the *Trichosporon* genus are responsible for superficial infections of the hair (white piedra) and skin (inguino-crural and perianal intertrigo), hypersensitivity pneumonitis (exclusively in Japan), and fatal deep infections. Superficial infections are frequently observed in immunocompetent patients, while deep infections are mainly documented in patients with hematological malignancies with profound neutropenia and in the immunocompromised [6].

Deep-seated but localized trichosporonosis with a favorable prognosis is possible in less debilitated populations (diabetics, prolonged corticosteroid therapy, peritoneal dialysis) [7].

In surgical contexts, *Trichosporon* infections are poorly documented, making this case particularly significant for medical literature. *Trichosporon spp.* is known to be a commensal organism of the digestive tract and can colonize the skin of healthy patients [8]. Although it is usually associated with infections in immunocompromised patients, there are reported cases of infections in immunocompetent individuals, especially after surgical interventions, as seen in our patient.

Regarding the origin of this abscess, colonization of our patient's skin by *Trichosporon spp.* is likely, especially in the context of broad-spectrum antibiotic therapy, which could be responsible for the infection if this agent has entered the retroperitoneal space. This hypothesis has also been raised by Baraboutis et al., who reported a case of mediastinitis and sternal osteomyelitis caused by *Trichosporon asahii* following a sternotomy [9]. Other etiologies have been reported in the literature, such as cross-contamination of surgical and anesthesia equipment [10].

The definitive diagnosis of deep trichosporonosis is essentially mycological. Microscopic examination of samples reveals blastospores with filaments and/or arthroconidia. On Sabouraud agar with or without actidione (cycloheximide), *Trichosporon spp.* colonies typically grow after an average of 7 days between 30 and 37 °C (but not at 45 °C). Macroscopically, these yeasts are creamy-white, rough, cerebriform, with a dome-shaped and irregular contour. Microscopic examination of colonies shows rounded or oval yeasts with true filaments and rectangular arthroconidia, along with some blastoconidia, typical of the *Trichosporon* genus [11].

Biochemically, these yeasts assimilate various carbohydrates and other carbon sources, but they do not possess fermentation capabilities. Another major characteristic of this genus is the ability to hydrolyze urea [1].

*Trichosporon spp*. have antigens in their wall similar to the polysaccharide capsule of *Cryptococcus neoformans*. Consequently, the search for cryptococcal antigen in serum tests may be positive in *Trichosporon* infections due to cross-reactivity with wall antigens [12]. Several authors have developed specific molecular biology techniques (PCR and nested PCR) that allow precise diagnosis of the *Trichosporon* genus and species. However, these techniques are not routinely applied in mycological diagnosis [13].

Unfortunately, the treatment of trichosporonosis remains a challenge because few studies have been dedicated to evaluating the in vitro and in vivo activities of different antifungals against clinically relevant *Trichosporon* species [4]. Nevertheless, the literature indicates that azoles, except for fluconazole, have good activities against *Trichosporon* species, while amphotericin B shows limited in vitro and in vivo activity against these microorganisms [8]. Our patient improved significantly under voriconazole, which has demonstrated superior in vitro activities against *Trichosporon* species compared to amphotericin B or fluconazole [14-16].

## Conclusions

*Trichosporon spp.*, although rare, should be considered as a potential pathogen in postoperative infections, especially in contexts where broad-spectrum antibiotics are used. This case also highlights the importance of a meticulous diagnostic approach, including mycological examination, to correctly identify unusual pathogens.

The significance of treatment in this case, facilitated by the precise identification of the pathogen and the targeted use of effective antifungals, such as voriconazole, highlights the importance of therapeutic management tailored to the sensitivity profiles of microorganisms.
